# Cost-Effective Reconstruction of the Anterior Chest Wall Following Resection of High-Grade Pleomorphic Sarcoma of the Sternum in an Adolescent: A Case Report

**DOI:** 10.7759/cureus.91696

**Published:** 2025-09-05

**Authors:** Dhanalakshmi Kadirvel, Srinath Ganesan, Ilango Parthasarathy, Gowtham Karthik V, Sairam KR

**Affiliations:** 1 General Surgery, Sree Balaji Medical College and Hospital, Chennai, IND; 2 Surgical Oncology, Sree Balaji Medical College and Hospital, Chennai, IND; 3 Surgical Oncology, Sri Ramachandra Institute of Higher Education and Research, Chennai, IND

**Keywords:** adolescent tumor, bone cement, chest wall reconstruction, cost-effective reconstruction, high-grade sarcoma, pleomorphic sarcoma, pmma block, polypropylene mesh, sternal resection, sternum tumor

## Abstract

A 15-year-old girl presented with a progressive, painless presternal swelling over two months. Contrast-enhanced CT revealed a heterogeneously enhancing solid mass arising from the mid-sternum with features suspicious for cortical destruction of the sternum. A core needle (Tru-Cut) biopsy confirmed high-grade pleomorphic sarcoma (Ki-67 ~70%, Vimentin and smooth muscle actin positive, S100 negative). The tumor was staged as Stage IIB (T2bN0M0, high grade, >5 cm, deep location) according to the American Joint Committee on Cancer soft tissue sarcoma staging system. Following neoadjuvant chemotherapy, the patient underwent wide local excision, including the body of the sternum and bilateral third and fourth costal cartilages/ribs. Chest wall reconstruction was performed using a polymethylmethacrylate cement block wrapped in polypropylene mesh and secured with the SternaLock system. Postoperative recovery was uneventful. This case highlights a cost-effective and structurally sound approach to sternal reconstruction using commonly available materials in resource-limited settings, with good oncologic and functional outcomes.

## Introduction

High-grade pleomorphic sarcoma of the sternum is a rare and aggressive malignancy requiring a multimodal management approach. Surgical resection with negative margins remains the mainstay of treatment for localized disease, particularly when involving the anterior chest wall or sternum [1]. Over the past few decades, the surgical management of chest wall tumors has evolved significantly, with advancements in techniques for both resection and reconstruction [2]. A thorough understanding of the anatomy, tumor behavior, and reconstruction materials is essential for optimal outcomes [3].

Historically, large sternal defects posed significant reconstructive challenges due to the sternum’s critical role in protecting mediastinal structures, maintaining chest wall stability, and preserving respiratory mechanics. Loss of sternal integrity can result in paradoxical chest wall motion (flail chest), impaired ventilation, and increased risk of infection or injury to underlying organs. Traditional reconstructive methods relied on soft tissue coverage alone, which provided protection but limited rigidity. To address this, innovative techniques such as the “rib-like” reconstruction approach were developed, where rigid prosthetic materials are contoured and fixed to mimic the structural support of native ribs, thereby restoring both stability and function [4]. More recently, advances in biomaterials and 3D printing technologies have enabled the creation of custom-designed implants tailored to the patient’s anatomy. These implants provide both structural strength and precise anatomical fit, making them particularly valuable in complex chest wall defects where conventional prostheses may not suffice [5].

## Case presentation

A 15-year-old previously healthy female presented to the surgical outpatient department with a progressively enlarging, painless swelling over the midline anterior chest wall for two months. There was no associated fever, trauma, constitutional symptoms such as weight loss or anorexia, or family history of malignancy. Clinical examination revealed a firm, non-tender, immobile swelling measuring approximately 5 × 5 cm over the mid-sternal region. The overlying skin was normal, and no regional lymphadenopathy was noted.

**Figure 1:**
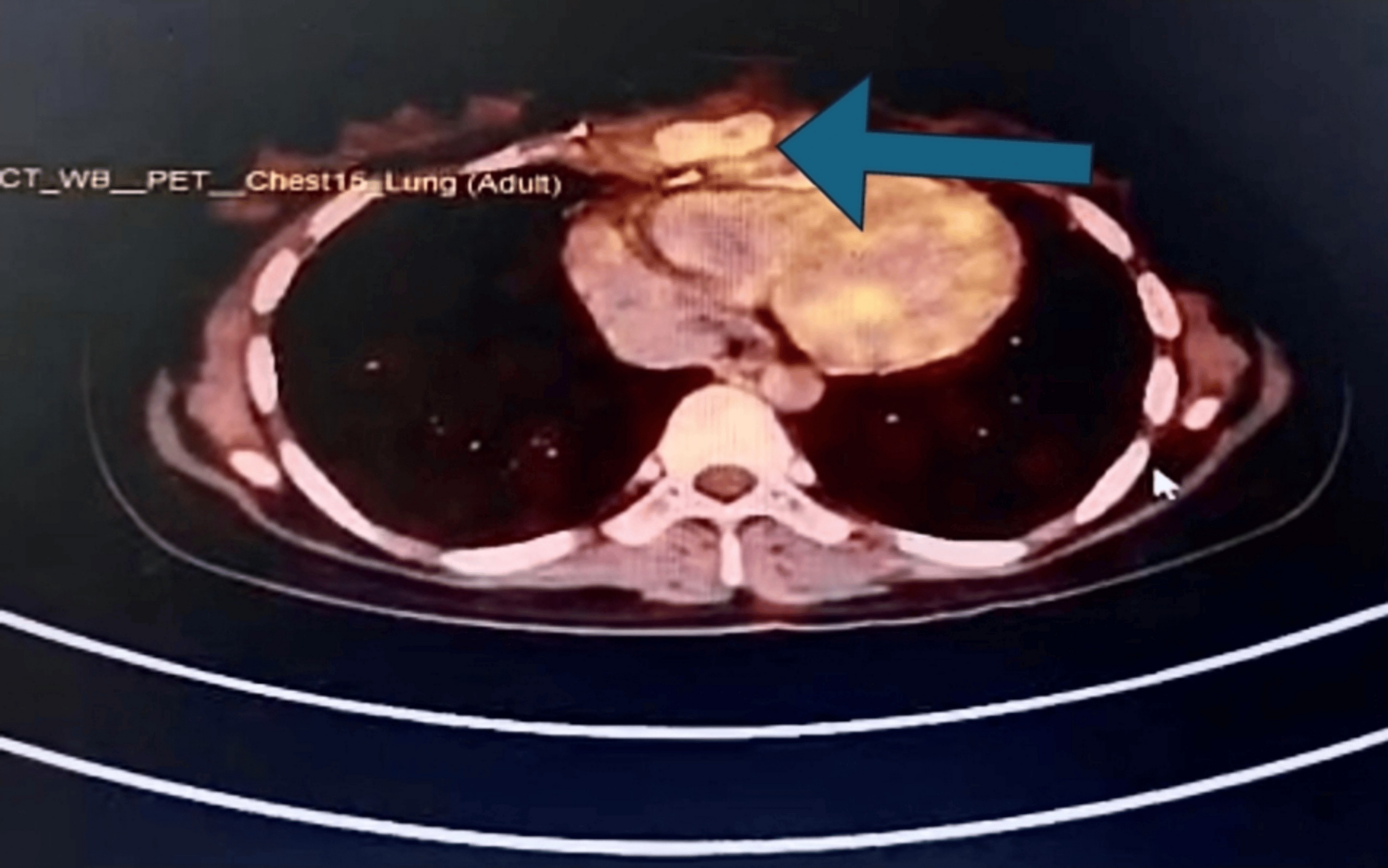
Axial PET-CT fusion image showing FDG-avid lesion in the mid-sternal region The image reveals a hypermetabolic, FDG-avid lesion involving the anterior mid-sternum, consistent with high-grade pleomorphic sarcoma. No evidence of intrathoracic or distant metastatic spread is seen. PET-CT: positron emission tomography-computed tomography, FDG: fluorodeoxyglucose

**Figure 2:**
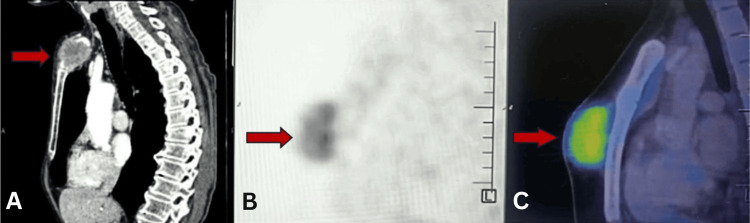
Sagittal PET-CT composite views demonstrating localized sternal tumor activity (A) CT alone, (B) PET alone, and (C) fused PET-CT sagittal images showing a metabolically active soft tissue lesion confined to the anterior chest wall overlying the sternum. The lesion exhibits high FDG uptake without adjacent organ invasion. PET: positron emission tomography, CT: computed tomography, FDG: fluorodeoxyglucose

An MRI of the thorax was performed, which revealed a 4.5 × 3.6 × 2.2 cm heterogeneously enhancing solid lesion in the deep subcutaneous plane of the anterior chest wall. The lesion extended posterolaterally, involving the left sternocostal junction and the adjacent third intercostal space, with partial involvement of the fourth intercostal space. Bony erosion of the mid-body of the sternum was suspected, and the lesion was seen abutting the bilateral third costal cartilages. To evaluate metastatic spread, an 18F-fluorodeoxyglucose (FDG) PET-CT was done, which demonstrated a well-defined FDG-avid lesion confined to the mid-body of the sternum without evidence of nodal or distant metastases (Figures 1-2). Based on these findings, the tumor was staged as Stage IIB, T2bN0M0, according to the American Joint Committee on Cancer soft tissue sarcoma TNM classification (tumor >5 cm, high-grade, deep, with no nodal or distant involvement).

A core needle biopsy was performed, and histopathology revealed a high-grade pleomorphic sarcoma. Immunohistochemistry (IHC) showed strong positivity for vimentin and smooth muscle actin (SMA), while S100 was negative. The Ki-67 proliferation index was approximately 70%, indicating a highly proliferative and aggressive tumor. These findings confirmed a diagnosis of non-metastatic high-grade pleomorphic sarcoma of the sternum. A summary of the diagnostic findings is provided in Table 1.

**Table 1:** Summary of diagnostic findings MRI: magnetic resonance imaging, PET-CT: positron emission tomography-computed tomography, IHC: immunohistochemistry, FDG: fluorodeoxyglucose, SMA: smooth muscle actin

Investigation	Findings
Clinical examination	5 × 5 cm firm, immobile, non-compressible, non-reducible, non-pulsatile mass with negative transillumination and normal overlying skin in midline
MRI thorax	4.5 × 3.6 × 2.2 cm enhancing mass, deep subcutaneous plane, bony involvement
PET-CT scan	FDG-avid mid-sternal lesion, no metastasis
Histopathology	High-grade pleomorphic sarcoma
IHC markers	Vimentin (+), SMA (+), S100 (–), Ki-67 ≈ 70%

In view of the aggressive nature of the tumor, the patient received four cycles of neoadjuvant chemotherapy with ifosfamide and docetaxel, administered at three-weekly intervals. A follow-up contrast-enhanced CT scan demonstrated partial regression, with the lesion reducing in size from 4.5 × 3.6 × 2.2 cm to 3.2 × 2.5 × 1.8 cm.

For definitive resection, the patient was taken up under general anesthesia in the supine position after obtaining informed and written consent from the patient’s guardian. A vertical midline incision extending from the suprasternal notch to the xiphoid process was made, elliptically incorporating the skin and subcutaneous tissue adherent to the tumor to ensure en bloc removal. Upon exposure, the lesion was observed to involve the mid-body of the sternum, the left sternocostal junction, and the adjacent third intercostal space, with partial extension into the fourth intercostal space, abutting the bilateral third costal cartilages.

Dissection was carried out in relation to vital structures. The retrosternal plane was developed by opening the endothoracic fascia, during which the bilateral pleurae were inadvertently breached. These were immediately identified and repaired intraoperatively, and bilateral intercostal drains were placed. The pericardium was carefully protected throughout.

**Figure 3:**
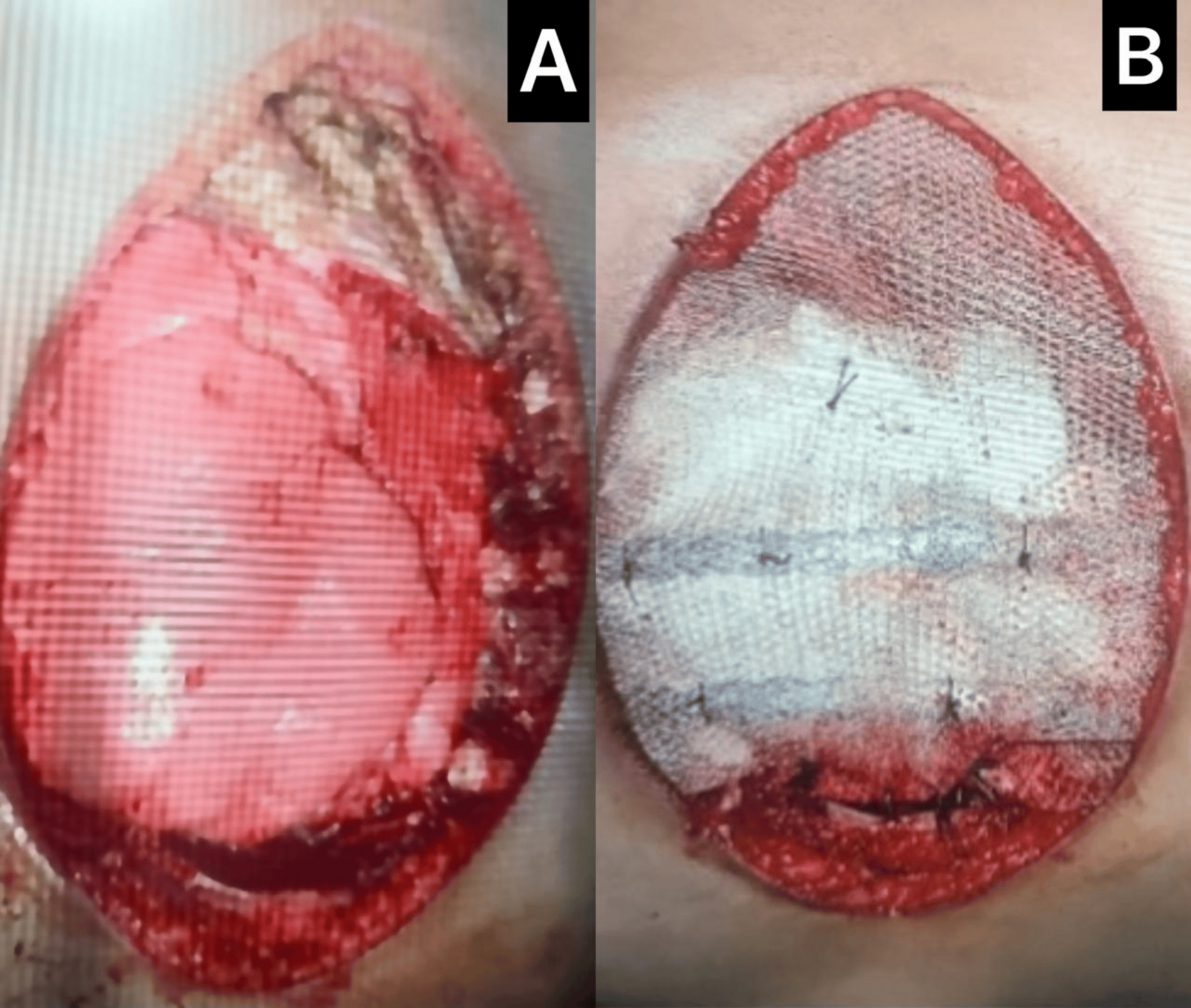
Intraoperative comparison of chest wall before and after neo-sternal reconstruction (A) Following en bloc resection of the tumor-involved sternum and adjacent ribs, the pericardium and underlying thoracic cavity are exposed. (B) The neo-sternum is reconstructed using a PMMA cement block wrapped in polypropylene mesh and anchored with titanium hardware, providing a stable and protective anterior chest wall. PMMA: polymethylmethacrylate

The manubrium sterni was transected using an oscillating saw, and the medial ends of the second, third, and fourth ribs were resected, approximately 3 cm on the left and 1 cm on the right, to obtain oncologically safe circumferential margins. The sternum was divided at the level of the fourth rib. The specimen, consisting of the mid-sternum, medial portions of bilateral second to fourth ribs, and surrounding soft tissues, was excised en bloc with adequate margins (Figure 3A). The resulting defect measured ~9 × 7 cm. Reconstruction was performed stepwise. First, the pericardium was shielded with a sheet of polypropylene mesh. Next, a neo-sternum was fashioned intraoperatively using polymethylmethacrylate (PMMA) bone cement, molded to the defect, and wrapped in polypropylene mesh. PMMA is a widely used, biocompatible, and moldable material with applications in cranioplasty and vertebroplasty. It has been reported as a cost-effective alternative to custom prostheses in chest wall reconstruction. This construct was anchored superiorly to the residual manubrium and laterally to the rib stumps (second to fourth ribs) using the SternaLock system (Figure 3B). The SternaLock system is a titanium plate-and-screw fixation device designed for rigid sternal fixation, adapted here to secure the prosthetic construct to native bone.

**Figure 4:**
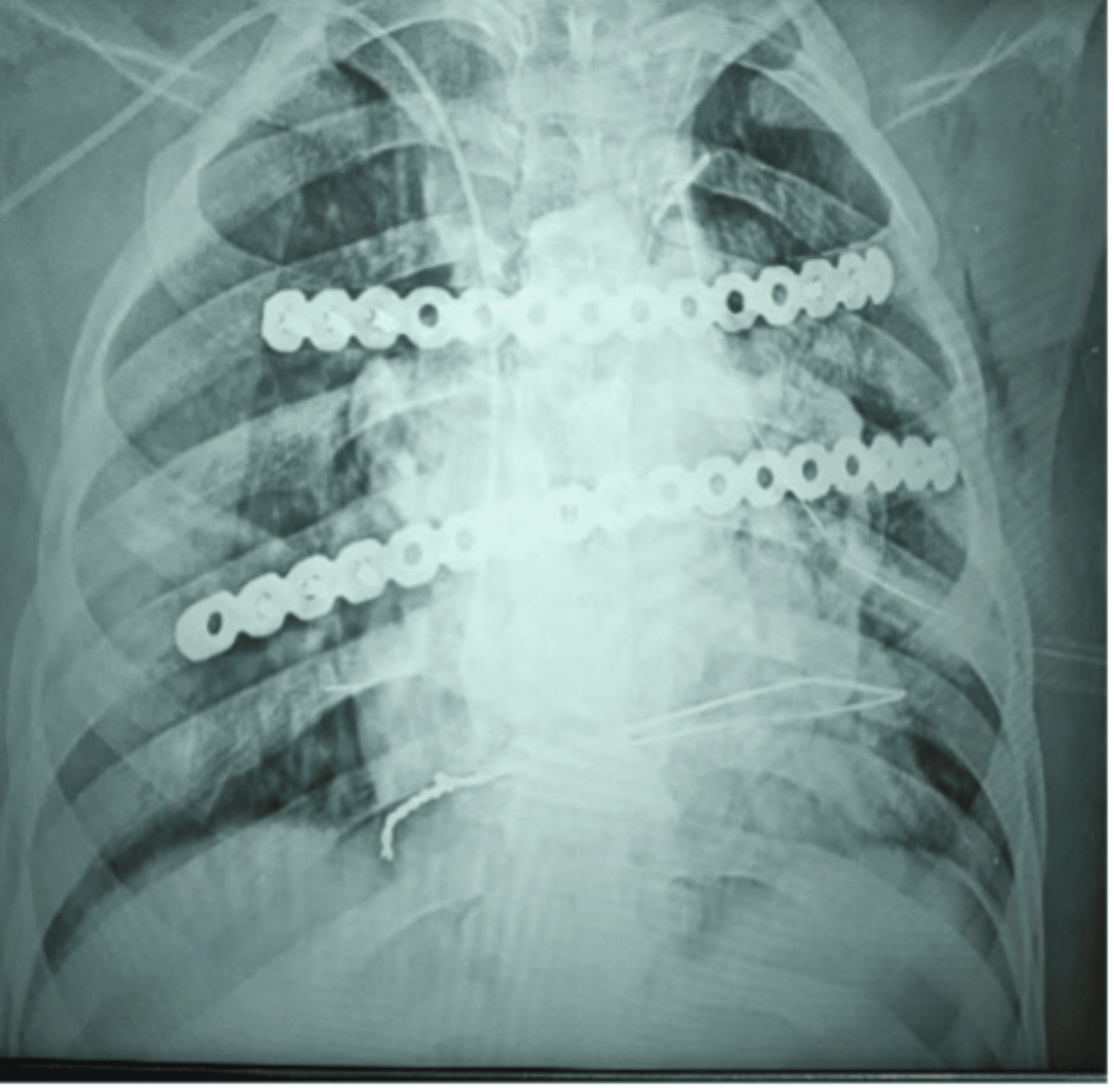
Immediate postoperative chest X-ray showing titanium plate fixation of neo-sternum and ribs Frontal chest radiograph revealing the position of the reconstructed sternum secured with dual horizontal titanium plates.Rib reconstruction and implant alignment are maintained.

Additionally, the second to fourth ribs bilaterally were reconstructed with rigid plate-screw fixation to restore continuity and provide added stability. Bilateral pectoralis major muscle flaps were mobilized and advanced medially to provide soft tissue coverage over the cement block. A Romovac suction drain was placed in the submuscular plane, and bilateral intercostal chest drains were inserted into the pleural cavities. Skin and subcutaneous tissues were closed in layers (Figure 4).

Table 2 outlines the key surgical steps and materials used for resection and reconstruction. A midline incision enabled exposure of the tumor and adjacent ribs. The incision was made through a vertical midline approach, incorporating the overlying skin and subcutaneous tissue adherent to the tumor in an elliptical fashion, to facilitate en bloc resection with safe margins. Postoperatively, the patient reported moderate incisional pain (VAS 5/10 on day one), which improved to 2/10 by day five and was managed with intravenous paracetamol and opioids initially, later transitioning to oral nonsteroidal anti-inflammatory drugs. Vital signs remained stable throughout. The wound was healthy without seroma, infection, or dehiscence, and daily chest radiographs confirmed satisfactory lung expansion.

**Table 2:** Surgical details PMMA: polymethylmethacrylate

Surgical step	Technique/material used
Incision	Midline vertical (6 cm)
Extent of resection	Mid-sternum + medial ends of 2nd to 4th ribs
Margins	3 cm (left) and 1 cm (right) from tumor edge
Pericardial protection	Polypropylene mesh overlay
Reconstruction material	PMMA bone cement block wrapped in polypropylene mesh
Fixation method	Titanium plate-screw system (SternaLock)
Rib reconstruction	Rigid plate fixation (2nd–4th ribs)
Soft tissue closure	Bilateral pectoralis major advancement flaps

The overall course was uneventful, and the patient tolerated the reconstructed chest wall well, with no cardiopulmonary or wound-related complications. She was ambulant and independent in activities of daily living at discharge on day seven, requiring no formal physical rehabilitation apart from incentive spirometry and routine chest physiotherapy. On follow-up at one, three, and six months, clinical examination and chest CT confirmed the mesh-PMMA construct remained stable without displacement or infection. The patient reported preserved quality of life, with normal breathing, coughing, and mobility, and did not experience any functional limitation or chest wall discomfort.

## Discussion

Sternal reconstruction following oncologic resection aims to achieve three primary objectives: restoration of chest wall stability, protection of intrathoracic organs, and preservation of respiratory mechanics. Recent advances in 3D custom implants, such as titanium prostheses and ribs fabricated through additive manufacturing, have transformed chest wall reconstruction. These patient-specific implants offer excellent anatomical conformity and biomechanical strength and have been successfully employed in both pediatric and adult populations [6,7]. However, their use is often limited in resource-constrained settings due to high costs, delayed availability, and logistical challenges.

In our patient, timely reconstruction was necessary following wide resection for high-grade sternal sarcoma to achieve oncologic control. Patient-specific titanium or PEEK implants were not feasible due to both resource constraints and the need for immediate surgery. We therefore opted for PMMA cement wrapped in polypropylene mesh, reinforced with titanium plating. This construct is inexpensive, readily available, and has been shown to provide sufficient rigidity, improved chest wall stability, and prevention of paradoxical movement [8]. The SternaLock system, initially designed for rigid fixation in sternal closure, has been adapted for use in chest wall reconstruction to anchor prosthetic materials, such as PMMA, thereby providing stability and facilitating early recovery [7,8].

Although rigid fixation has been debated, most evidence supports its role in reducing respiratory compromise and facilitating early mobilization, particularly in younger patients [9,10]. Our case demonstrated that combining PMMA with titanium plating achieved adequate stabilization and protection of mediastinal structures with good early functional outcomes.

Histopathologic evaluation confirmed a high-grade soft tissue sarcoma based on the FNCLCC (Fédération Nationale des Centres de Lutte Contre le Cancer) grading system, with a score of grade III, which carries a poorer prognosis compared to low- or intermediate-grade tumors. Negative margins were achieved, an important determinant of local control and survival [11,12]. Prognostic factors such as tumor grade, resection margin, and histological subtype remain central in predicting outcomes after sternal tumor resection.

For soft tissue coverage, muscle flaps such as the pectoralis major or latissimus dorsi have been widely recommended [13]. In our patient, we achieved adequate coverage with mobilized pectoralis flaps to protect the prosthesis and reduce the risk of exposure or infection. To mitigate the limitations of PMMA, particularly its exothermic polymerization reaction, cement was allowed to partially polymerize ex vivo before placement, thereby minimizing thermal injury to adjacent tissues. Additionally, vascularized flap coverage and strict aseptic technique were employed to reduce the risk of infection, which remains a theoretical concern with non-biologic materials.

Radiation-induced sarcomas, particularly following breast cancer treatment, are well-documented in the literature and highlight the need for long-term vigilance in patients with prior oncologic therapy [14,15]. While breast cancer radiation regimes are indeed being de-escalated to lower doses and shorter durations in modern practice, the association between radiotherapy and secondary sarcoma development remains clinically relevant, especially in older treatment cohorts. Thus, this context remains important when evaluating similar patients.

Table 3 provides a comparative analysis of common sternal reconstruction techniques, including their advantages and limitations:

**Table 3:** Comparison of chest wall reconstruction techniques after sternal resection 3D: three dimensional, PMMA: polymethylmethacrylate

Reconstruction method	Material	Advantages	Limitations	References
3D-printed titanium implants	Custom titanium ribs/prostheses	Anatomical fit, rigidity, ideal for complex defects	High cost, limited accessibility	[5-7]
PMMA cement with mesh	PMMA + polypropylene	Low-cost, customizable intraoperatively	Non-biologic, potential infection risk	[1,3,9]
Titanium mesh + rigid fixation	Preformed plates, often with mesh	Structural support, biocompatibility	Less flexible, technically demanding	[4,10]
Muscle flap reconstruction	Pectoralis major, latissimus dorsi	Vascularized coverage, reduced infection risk	No bony stability, donor site morbidity	[8,13]
Composite techniques	Titanium plate + mesh + flap	Functional + aesthetic restoration	Requires multidisciplinary expertise	[9,10,12]

For young patients with high-grade sternal sarcomas, complete oncologic resection with negative margins remains the cornerstone of treatment. Reconstruction should be individualized based on resource availability and patient factors. Our case demonstrates that a PMMA-polypropylene mesh construct, reinforced with titanium plating and adequately covered with vascularized flaps, offers a reliable and accessible solution where patient-specific 3D implants are not feasible. This strategy provided satisfactory chest wall stability, protected mediastinal structures, and preserved respiratory mechanics, yielding excellent early outcomes.

## Conclusions

Sternal reconstruction following oncologic resection in adolescents poses unique technical and psychosocial challenges. Our case illustrates a cost-effective and technically feasible reconstruction using PMMA bone cement and mesh, resulting in stable thoracic integrity and a favorable postoperative outcome. This approach is especially valuable in resource-constrained settings and supports wider adoption in similar clinical scenarios.
